# Histopathology of the exanthema in DRESS in correlation with markers of severe systemic involvement

**DOI:** 10.1186/2045-7022-4-S3-O8

**Published:** 2014-07-18

**Authors:** Margarida Gonçalo, Inês Coutinho, Miguel Gouveia, Rita Cabral, Neide Pereira, José Carlos Cardoso, Óscar Tellechea

**Affiliations:** 1 Clinic of Dermatology, Faculty of Medicine, University of Coimbra, University Hospital, Coimbra, Portugal

## Background

Exanthema in DRESS is considered to have no specific clinical features similarly to histopathology, performed systematically in a few studies. Nevertheless, histologic findings may be related with systemic symptoms.

## Objective

We characterized histopathologic findings in DRESS and correlated them with the severity of the systemic involvement, the culprit drug or HHV-6 reactivation.

## Methods

Skin biopsies performed in in-patients with a diagnosis of DRESS according to REGISCAR criteria, were independently evaluated by two Dermatopathologists. Epidermal and dermal parameters were scored (lymphocyte, eosinophil and neutrophil infiltrate, lymphocyte exocytosis, spongiosis and necrotic/vacuolated keratinocytes) and correlated with the severity of systemic symptoms (eosinophilia, liver cytolysis and cholestasis, evaluated respectively by serum ALT and GGT values) and the presence of circulating HHV-6 DNA.

## Results

In 15 patients (9M/6F, mean age 53.3y) with DRESS mainly from allopurinol (8) or anticonvulsants (5), we observed an exfoliative erythroderma (3) or maculopapular exanthema (12), with facial edema (9), lip/oral erosions (2), atypical targets (2), flaccid bullae (1), purpura (2) and pustules (2). Histopathology showed, in variable intensity and proportions, a lymphocyte and/or eosinophil dermal infiltrate, lymphocyte exocytosis, spongiosis, necrotic/vacuolated keratinocytes, combining in a more eczematous, lichenoid, erythema multiforme-like or pseudolymphomatous-like reaction. There was a significant positive correlation between the score of lymphocyte infiltration and the severity of hepatic cytolysis and circulating eosinophils (for both, Pearson' coefficient r=0.51, p<0.05), but no correlation between the score for epidermal damage (keratinocyte vacuolization/necrosis) and systemic involvement, or correlation with the culprit drug or viral reactivation.

## Conclusions

Severity of liver cytolysis was correlated with the intensity of the dermal infiltrate, but not with the intensity of epidermal damage, as previously described. Although DRESS histopathology cannot be used as a diagnostic tool it can be an additional prognostic marker, but more studies are needed. There were clinic and, also, histologic aspects common to SJS/TEN (bullous forms with epidermal cytolysis) and maculopapular exanthema, suggesting possible overlapping patterns between DRESS and these drug eruptions.

